# Author's reply

**DOI:** 10.1192/bjb.2021.120

**Published:** 2022-02

**Authors:** Norman Poole

**Affiliations:** Independent Researcher, UK. Email: norman.poole@gmail.com

I thank Dr Margaret White for the letter in response to my recent editorial ‘Publishing controversy’. It raised important challenges. Why have the trans health papers been published online and in print even though written by non-specialists in gender identity, and should opposing views be relegated to the Correspondence section? First, all papers published online also appear in the paper journal eventually. To do otherwise would have marked these papers out as somehow different. Dr White does not wish to silence debate, but not publishing in print form as usual would be a form of censure even if not censor. Although this is a controversial and contested area, the papers did not express extreme views. In fact, Marci Bowers, president-elect of the World Professional Association for Transgender Health, recently raised concerns similar to those expressed in the *Bulletin* papers. However, we remain keen to present all opinions so have commissioned papers from gender identity experts, which are making their way through the editorial process. When the papers by Griffin et al and Evans were published on First View, they quickly attracted several complaints with demands for their retraction, which as explained in the editorial was not appropriate. Those authors were invited to write opposing articles but unfortunately, for their own reasons, none took up the offer. Hence our decision to publish all the available letters alongside the original papers so readers can evaluate the arguments for themselves. I hope this and the forthcoming papers assures Dr White that no one's voice is relegated to the correspondence section in the *BJPsych Bulletin*, but letters, such as Dr White's, are also an invaluable element of discourse.

